# Genome-Wide Identification and Analysis of the Class III Peroxidase Gene Family in Tobacco (*Nicotiana tabacum*)

**DOI:** 10.3389/fgene.2022.916867

**Published:** 2022-06-13

**Authors:** Lingtong Cheng, Lanxin Ma, Lijun Meng, Haihong Shang, Peijian Cao, Jingjing Jin

**Affiliations:** ^1^ China Tobacco Gene Research Center, Zhengzhou Tobacco Research Institute of CNTC, Zhengzhou, China; ^2^ Zhengzhou Research Base, State Key Laboratory of Cotton Biology, School of Agricultural Sciences, Zhengzhou University, Zhengzhou, China

**Keywords:** plant peroxidases, tobacco, expression pattern, 3D model, stress

## Abstract

Class III peroxidases (PODs) are plant-specific enzymes that play significant roles in plant physiological processes and stress responses. However, a comprehensive analysis of the POD gene family in tobacco has not yet been conducted. In this study, 210 non-redundant POD gene members (*NtPODs*) were identified in tobacco (*Nicotiana tabacum*) and distributed unevenly throughout 24 tobacco chromosomes. Phylogenetic analysis clustered these genes into six subgroups (I-VI). Gene structure and motif analyses showed the structural and functional diversity among the subgroups. Segmental duplication and purifying selection were the main factors affecting *NtPOD* gene evolution. Our analyses also suggested that *NtPOD*s might be regulated by miRNAs and cis-acting regulatory elements of transcription factors that are involved in various biological processes. In addition, the expression patterns in different tissues and under various stress treatments were investigated. The results showed that the majority of *NtPODs* had tissue-specific expression patterns and may be involved in many biotic and abiotic responses. qRT-PCR analyses of different tissues and stress treatments were performed to verify transcriptome patterns. Expression of a green fluorescent protein-NtPOD fusion confirmed the plasma membrane localization of NtPOD121 and NtPOD4. Furthermore, 3D structures provided evidences of membrane-bound peroxidase. These findings provide useful information to better understand the evolution of the *NtPOD* gene family and lay the foundation for further studies on POD gene function in tobacco.

## Introduction

Peroxidases (EC 1.11.1.X) are a large family of enzymes that are widely distributed in living organisms and catalyze the oxidation of various substrates with hydrogen peroxide (H_2_O_2_) as an electron acceptor (Welinder, 1992; Kidwai et al., 2020). Peroxidase performs three distinct cycles: the peroxide cycle, the oxidation cycle, and the hydroxyl cycle, leading to the elimination and generation of reactive oxygen species (ROS) (H_2_O_2_, O_2_^−^, and OH^−^) (Kidwai et al., 2020). Therefore, peroxidase can be considered as a bifunctional enzyme maintaining intracellular ROS levels by oxidizing various substrates with H_2_O_2_ or generating ROS (Passardi et al., 2004). According to their structure, they can be divided into heme or non-heme containing proteins. The heme-containing peroxidases can be further subdivided into animal and non-animal groups (Mathé et al., 2010). Previous study contended that non-animal peroxidases contain three large families: class I, II, and III (Cosio and Dunand, 2009). Class III peroxidases (guaiacol peroxidases, EC 1.11.1.7) are heme oxidoreductase enzymes that exist in various plants as a multigenic family (Mathé et al., 2010). In previous studies, different abbreviations, such as POX, Px, PER, POD, and Prx, have been used for class III peroxidases (e.g.) (Almagro et al., 2009).

Class III peroxidases are plant-specific glycoproteins and have been extensively studied in higher plants. PODs have been shown to play a role in a broad range of physiological and developmental processes, including cell wall metabolism, lignification, auxin metabolism, stress tolerance, and defense response (Pandey et al., 2017). To date, class III peroxidases have been investigated and characterized in a variety of plant species, including *Arabidopsis* (Tognolli et al., 2002), cassava (Wu et al., 2019), Chinese pear (Cao et al., 2016), maize (Wang Y et al., 2015), rice (Passardi et al., 2004), orange (Li et al., 2020), grapevine (Xiao et al., 2020), and soybean (Aleem et al., 2022). Some genetic evidences suggested that class III peroxidase is an abiotic stress-responsive enzyme in different plant species. For example, the overexpression of *AtPrx64* could enhance the tolerance of tobacco to aluminum stress (Wu et al., 2017), and the overexpression of *TaPRX-2A* in wheat exhibited an enhanced tolerance to salt stress (Su et al., 2020). Similarly, the overexpression of *CrPRX,* a *Catharanthus roseus* peroxidase, improved tolerance of tobacco to salt and drought stress (Jaggi et al., 2011). Peroxidases also play a role in resistance to pathogens. It has been reported that *TpoxN1* expression was induced in tobacco within 20 min of wounding, and the response could be sustained for longer than 2 weeks, indicating that *TpoxN1* may involve in wounding-healing (Sasaki et al., 2002). The overexpression of *CaPO2* in *Arabidopsis* enhanced the accumulation of H_2_O_2_, the expression of many pathogen-responsive genes, and resistance to *Xanthomonas campestris* pv*. vesicatoria* (Choi and Hwang, 2012). Hence, plant PODs may play an essential role in the response to biotic and abiotic stresses.

Tobacco (*Nicotiana tabacum*) is an important commercial crop in the world and is often used as a model organism to conduct plant genetic researches. During growth, tobacco is often affected by adverse stresses, including pathogenic bacteria, drought, cold, salinization, and heavy metals (Dana et al., 2006; Wei et al., 2008). However, the POD gene family in tobacco remains unexplored. In this study, we conducted a comprehensive analysis of the structure and function of class III peroxidases in tobacco, including phylogenetic relationships, duplication events, functional domain characterization, and cis-acting elements. The 3D structures of eight PODs were explored to elucidate their mechanisms. Additionally, the expression patterns in eight typical tissues and in response to different abiotic and biotic stresses were investigated. Our results provide comprehensive insights into the biological functions of class III peroxidases in tobacco plants.

## Materials and Methods

### Identification of the Peroxidases Gene Family in the Tobacco

To identify POD genes in tobacco, the protein sequences of 73 *Arabidopsis* (Tognolli et al., 2002) and 138 rice PODs (Passardi et al., 2004) were searched in the Sol Genomics Network (Fernandez-Pozo et al., 2015) and our unpublished tobacco genome using the TBLASTN (v2.12.0) (McGinnis and Madden, 2004) program with default parameters. HMMER (v3.3.2) (Finn et al., 2011) was employed to identify the candidate tobacco POD genes using the Hidden Markov Model (HMM) based on the POD conserved domain (PF00141). All candidate sequences were confirmed using PFAM (http://pfam.xfam.org/) and SMART (http://smart.embl-heidelberg.de/). The physicochemical properties of the POD proteins, including molecular weight (MW) and isoelectric points (pI), were predicted using the online ExPASy tool (http://web.expasy.org/protparam) (Gasteiger et al., 2003).

### Phylogenetic, Gene Structure, and Conserved Motif Analyses

A phylogenetic tree of the *NtPOD* genes was constructed using the neighbor-joining (NJ) method with a bootstrap value of 1,000 in MEGA X (Kumar et al., 2018). To reveal the exon-intron organization of the POD genes in tobacco, TBtools (v1.09867) (Chen et al., 2020) were used to determine the gene structures of each *NtPOD* gene. The motifs were analyzed using the MEME program (http://meme-suite.org/tools/meme). The maximum number of motifs was set to 10, the optimum width of motifs was set to 6–200 amino acid residues, and the remaining settings were kept at default values (Bailey et al., 2015). Subsequently, the identified motifs were annotated using InterProScan (http://www.ebi.ac.uk/Tools/pfa/iprscan/) (Quevillon et al., 2005).

### Chromosomal Location and Gene Duplication Analyses

All *NtPOD* genes were mapped to their respective chromosomes. Chromosome maps of all identified *NtPODs* were drawn using TBtools (v1.09867) software (Chen et al., 2020). For gene duplication analysis, the amino acid sequences of *NtPODs* were aligned using BLAST (v2.12.0, e-value = 1e^−5^) (McGinnis and Madden, 2004). Gene pairs were considered as duplicated only if their similarity was ≥90%. Duplicated gene pairs located adjacent to the same chromosome were defined as tandemly duplicated, whereas those positioned on separate chromosomes were defined as segmental duplicates. The non-synonymous (*Ka*) and synonymous substitution (*Ks*) rates of duplicated *NtPOD* genes were calculated using ParaAT2.0 program (Zhang et al., 2012). Furthermore, the *Ka/Ks* ratio was calculated to evaluate selection pressure.

### Promoter Analysis and MiRNA–*NtPOD* Interaction Prediction

The 1,500 bp upstream sequence from the transcription start site was used to analyze the cis-elements. Putative stress or hormone-responsive cis-elements in the promoter region of the *NtPOD* genes were obtained using PlantCARE (http://bioinformatics.psb.ugent.be/webtools/plantcare/html/) (Lescot et al., 2002). Mature tobacco miRNAs were downloaded from the miRBase database (Kozomara et al., 2019). The regulatory relationship between miRNAs and *NtPODs* were searched using PsRNATarget (http://plantgrn.noble.org/psRNATarget/) (Dai and Zhao, 2011) with the default settings. The regulatory relationship between *NtPODs* and transcription factors was retrieved from the Plant Transcription Factor Database PlantTFDB (http://planttfdb.cbi.pku.edu.cn) (Jin et al., 2016) (specie setting with *Nicotiana tabacum*) by searching for promoter sequences. Cytoscape (Smoot et al., 2011) was used to visualize the interaction networks.

### Expression Analysis

Transcriptome data from eight distinct tissues (leaves, veins, blades, stems, roots, callus, axillary buds, and seeds) of tobacco were used to investigate the expression patterns of the *NtPOD* genes. The sampling stages for each tissue are listed in Supplementary Table S1. Raw sequence data of different tissues were obtained from PLncDB **(** http://plncdb.tobaccodb.org/) (Jin et al., 2021) and our unpublished data. Raw RNA-seq datasets, including cold (Jin et al., 2017), drought (Yang H et al., 2017), cadmium (Cd) (He et al., 2016), topping (Chen et al., 2019), NaCl, abscisic acid (ABA) (Wu et al., 2021), cucumber mosaic virus (CMV) (Liu et al., 2019), and *Phytophthora nicotianae* (Yang J. K et al., 2017) were obtained from the SRA database (Leinonen et al., 2010). All clean reads were mapped to the tobacco reference genome using Hisat2 (2.2.1) (Kim et al., 2019). The FPKM values were computed using the StringTie software (2.1.7) (Pertea et al., 2015).

### Plant Growth Conditions and Stress Treatments

The cultivated tobacco variety K326 was used to analyze the expression of POD genes in various tissues and stress treatments. The seedlings were cultivated in plastic pots with a 16 h light photoperiod at 28°C during the day and at 23°C at night. Plant root, stem, leaf, vein, axillary bud, terminal bud, flower, and seed samples were collected as described in our previous study (Wang Z et al., 2015). For salinity stress, the tobacco seeds were first germinated and cultivated in plastic pots under normal conditions for 14 days, and then exposed to salt (150 mM NaCl) for 7 days (Cheng et al., 2009). After 1 month of germination, the tobacco seedlings were treated for 6 h with plant hormones, including ABA (10 µM), IAA (10 µM) (Braybrook, 2017), salicylic acid (SA) (10 µM) (Kawano and Muto, 2000), and jasmonic acid (JA) (10 µM). Untreated plantlets were used as the control (CK). The treated and control plantlets were collected after treatment, and then all the samples were immediately frozen in liquid nitrogen and stored at −80°C.

### RNA Isolation and QRT-PCR

A SuperPure Plantpoly RNA Kit (Gene Answer, Beijing, China) was used to extract total RNA from the plant samples. DNA contamination was eliminated by digestion with RNase-free DNase I (Gene Answer). Reverse transcriptase M-MLV (Takara Biomedical Technology, Beijing, China) was used to synthesize first-strand cDNA using 1 μg of total RNA as a template. The cDNA was diluted to 50 ng/µl. RT-PCR was performed using a SYBR Green kit (Imagene, Beijing, China) in a 20-μl reaction solution. The PCR program was as follows: 95°C for 30 s, 40 cycles of 95°C for 10 s, and 60°C for 30 s. The expression levels of target genes were standardized to the expression level of the *NtL25* (Volkov et al., 2003) gene using the 2^−∆∆Ct^ method. Three independent biological replicates were used for each gene. The gene specific primers used for qRT-PCR are listed in Supplementary Table S2

### Subcellular Localization Analysis and 3D Structure Predictions

Signal peptide sequence and potential cleavage site of NtPODs were conducted by SignalP 5.0 program (Almagro Armenteros et al., 2019). The transmembrane domains of POD proteins were analyzed by the online tool TMHMM server v2.0 program (http://www.cbs.dtu.dk/services/TMHMM/) (Krogh et al., 2001), and their subcellular localization were predicted by WoLF PSORT (https://www.genscript.com/wolf-psort.html) (Horton et al., 2007). To verify subcellular localization of NtPODs, we generated C-terminal green fluorescent protein (GFP) fusions for two POD proteins and visualized their subcellular location by confocal microscopy after transient expression of the fusions in *Nicotiana benthamiana*. The three-dimensional (3D) structures of NtPOD proteins were predicted using AlphaFold2 (Jumper et al., 2021), and displayed using PyMOL software (http://pymol.org/) (DeLano, 2002). The quality of the predicted 3D structures was measured using ERRAT test scores on the SAVES server (https://saves.mbi.ucla.edu/). Molecular docking was performed using AutoDock Vina (Trott and Olson, 2010).

## Results

### Genome-Wide Identification of Peroxidases in Tobacco

Based on 73 PODs from *Arabidopsis* and 138 PODs from rice, we used BLAST and HMMER to search for PODs against the tobacco genome. A total of 210 non-redundant *NtPODs* were identified as class III peroxidases, denoted as *NtPOD1* to *NtPOD210* (Supplementary Table S3). The length of NtPOD proteins ranged from 250 (NtPOD116) to 500 (NtPOD26) amino acid residues, with an average of 326 amino acids (Supplementary Table S4). The relative molecular weight varied from 27.45 (NtPOD11) to 54.50 kDa (NtPOD26), with isoelectric points ranging from 4.57 (NtPOD192) to 10.03 (NtPOD138) (Supplementary Table S4, Supplementary Figure S1).

### Phylogenetic, Gene Structure, and Conserved Motif Analyses of *NtPOD* Gene Family

**FIGURE 1:**
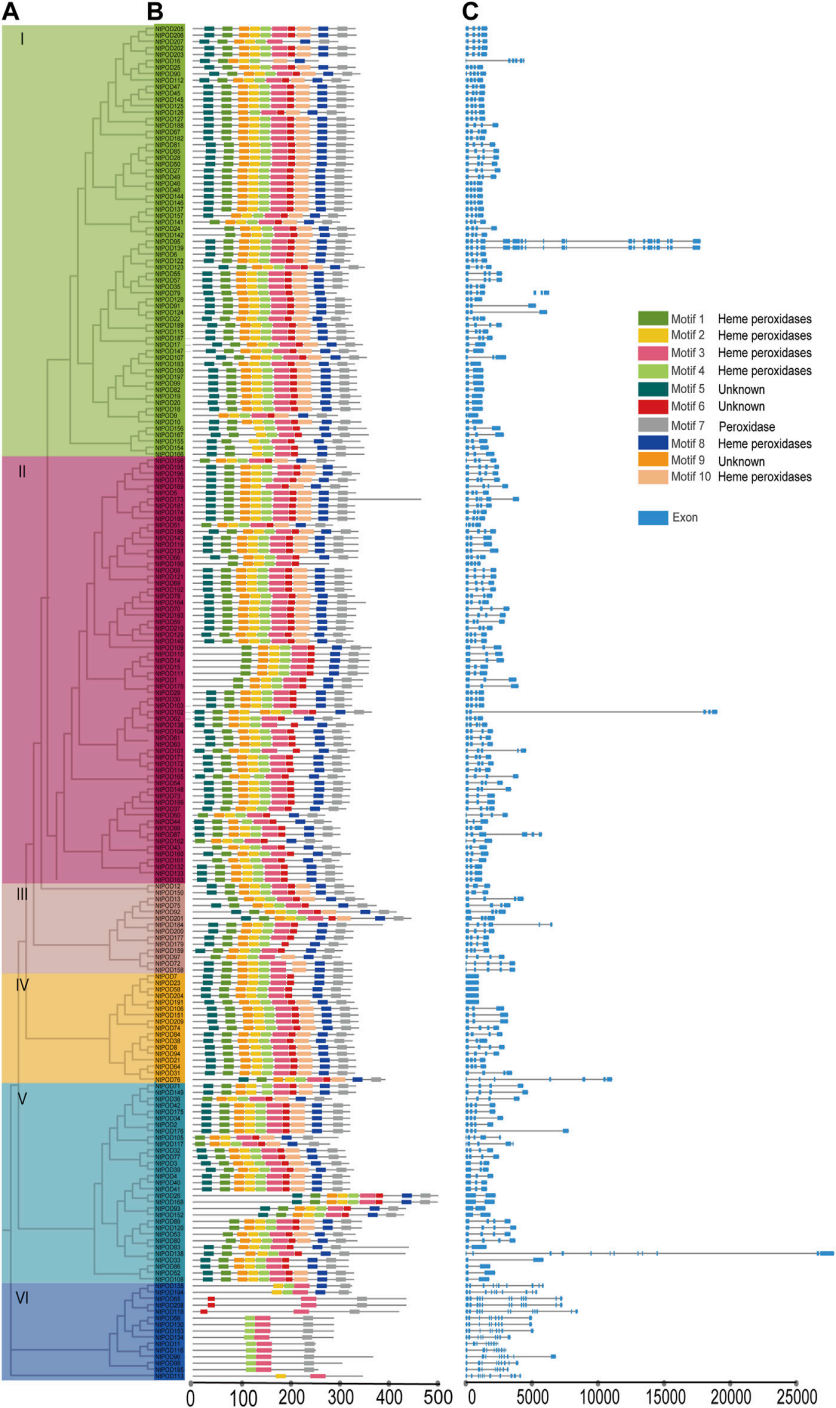
Phylogenetic tree, conserved motif, and gene structure of *NtPOD* genes. **(A)** Phylogenetic relationship among the *NtPOD* genes based on the amino acid sequence alignment. **(B)** Conserved motifs in amino acid sequence of different sub-groups (I-VI) of NtPODs. The 10 different colored boxes on the right represent diverse conserved motifs. The detailed sequences of motifs are listed in Supplementary Table S5 **(C)** Exon-intron analysis of six subgroups of *NtPODs*.

Phylogenetic analysis revealed that *NtPOD* genes could be divided into six subgroups (Figure 1A). Large subgroups I and II consisted of 67 and 66 *NtPOD* members, respectively, whereas small subgroups III, IV, and VI contained 14, 17, and 15 *NtPOD* members, respectively. Subgroup V comprised of 31 *NtPOD* members. The ten most conserved motifs for NtPODs were explored using the MEME program and annotated using InterProScan (Supplementary Table S5). Seven motifs (1, 2, 3, 4, 7, 8, and 10) were annotated as peroxidase domains, which were present in most of the NtPODs (92.4%, 94.3%, 99.0%, 98.1%, 98.6%, 93.3%, and 62.86%) (Figure 1B), suggesting that these motifs have been preserved for a long time. In total, 189 NtPODs in subgroups I-V contained at least seven motifs (1, 3, 4, 6, 7, 8, and 9), except for NtPOD16 (in subgroup I), NtPOD190 (in subgroup II) and NtPOD179, NtPOD97, NtPOD72, NtPOD158 (in subgroup III), (Figure 1B). Interestingly, subgroup VI was distinct from the other groups, and most members contained only motifs 3, 4, and 7 (Figure 1B). Furthermore, some unknown motifs (5, 6, and 9) were found in some subgroups.

To obtain further insights into POD evolution, we examined the exon-intron structures of the *NtPOD* genes. The results demonstrated structural variation among these *NtPOD* genes, ranging from 1 to 12 exons, whereas most *NtPODs* contained four exons and three introns (Figure 1C). Among the *NtPOD* genes, 50.0% (105/210) consisted of four exons, and 58.2% of subgroup I (39/67) had four exons (Figure 1C). Subgroup VI was exon-rich with 9–12 exons, whereas subgroup IV contained fewer exons (between one and four) (Figure 1C). Generally, *NtPOD* genes in the same subgroup exhibited similar exon-intron features, providing further evidence of their phylogenetic relationships.

### Distribution and Duplication of *NtPOD* Gene Family

**FIGURE 2:**
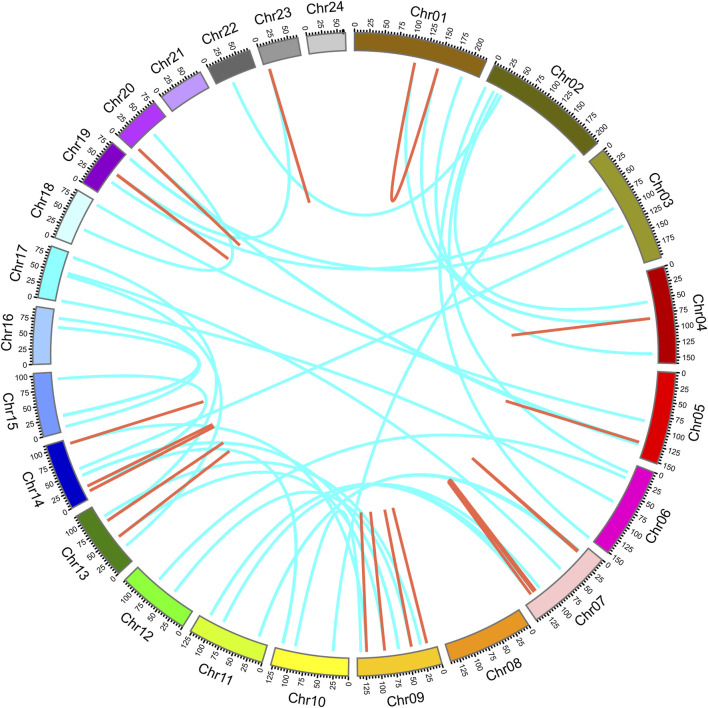
Distribution of tandem and segmental duplicated POD genes in the tobacco genome generated by Circos. Tandem and segmental duplicated gene pairs are linked by the red in same chromosome and cyan lines between chromosomes, respectively.

To explore the distribution of *NtPODs*, a physical map was constructed using TBtools. In total, 155 *NtPODs* were mapped onto tobacco chromosomes, while the others were mapped onto scaffolds (Supplementary Figure S2). Notably, there was no POD gene distribution on chromosomes 21 or 24. The *NtPOD* genes were unevenly distributed throughout the 22 chromosomes (Supplementary Figure S2). Gene duplication is an important mechanism for the evolution of novel gene functions. Segmental and tandem duplications are considered as the two major mechanisms of gene family expansion in plants. To further investigate the expansion of POD genes in tobacco, we aligned the nucleotide sequences of *NtPOD* genes to identify duplication events (Figure 2). Finally, 103 duplication events consisting of 109 paralogs were identified, including 25 tandem duplications and 78 segmental duplications. Similar to previous findings (Cai et al., 2021; Meng et al., 2021), segmental duplication was also 2-3-fold higher than tandem duplication in the *NtPOD* family, which was indicative of its contribution to the evolution and expansion of the *NtPOD* family.

Next, using the non-synonymous (*Ka*) and synonymous (*Ks*) ratios, we attempted to understand the evolutionary selection for the duplicated *NtPOD* genes (Supplementary Table S6). In general, a ratio of *Ka/Ks* greater than 1 indicates positive selection, while a ratio of *Ka/Ks* less than 1 indicates purifying selection and *Ka/Ks* equal to 1 indicates neutral selection. Among the 103 duplicated events, we found that most *Ka/Ks* ratios were less than 0.47 (Supplementary Table S6 and Supplementary Figure S3), indicating that these genes underwent purifying selection during evolution. Positive selection was observed in only two duplication events (*NtPOD112*/*NtPOD90* and *NtPOD90*/*NtPOD25*) (Supplementary Table S6).

### Cis-Acting Elements and Regulation Networks for *NtPODs*

**FIGURE 3:**
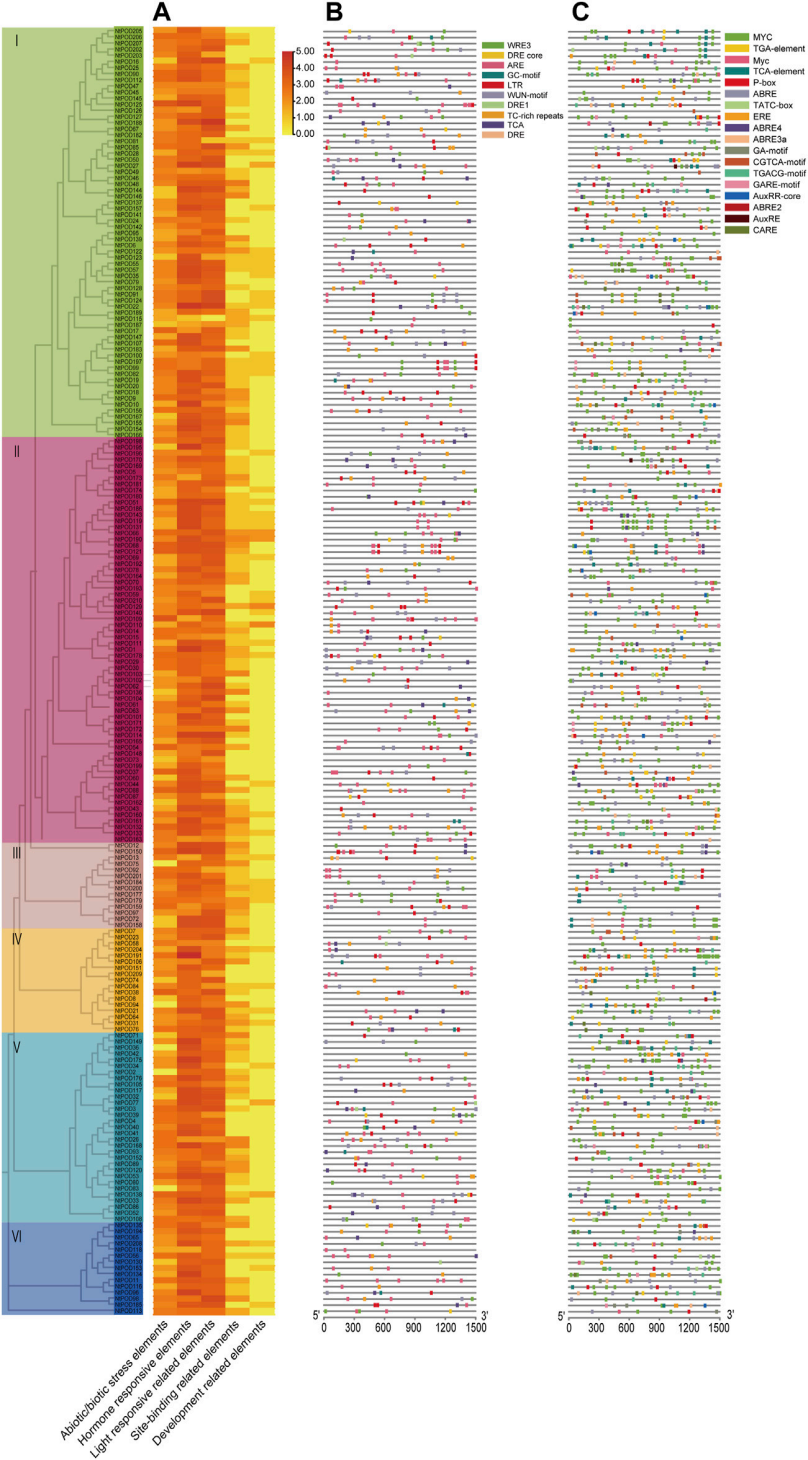
Prediction of cis-acting elements in the promoter sequences of *NtPOD* genes. **(A)** The number (log_2_ (count)) of cis-acting elements detected in the promoter region of each *NtPOD* gene. **(B)** Distribution of abiotic/biotic stress-related elements in *NtPODs*. **(C)** Distribution of hormone-responsive elements in *NtPODs.*

The upstream promoter regions of genes possess many cis-acting elements that can regulate gene expression. To better understand the potential regulatory mechanisms of *NtPOD* genes, we identified the presence of cis-elements in the promoter regions of *NtPOD* genes. The identified cis-acting elements were further classified into five distinct groups, based on their putative functions (Figure 3 and Supplementary Table S7). The most abundant elements were light-responsive elements, including Box 4, TCT-motif, and GT1-motif. Regulatory elements related to abiotic and biotic stresses were also found to be abundant, comprising 10 cis-elements, including anoxic, cold, anaerobic, drought, disease, and wound responsive elements. A total of 155 *NtPOD* genes contained cis-acting elements involved in the anaerobic induction (ARE) (Figure 3 and Supplementary Table S7). Hormone-responsive elements included 19 members, most of which were associated with ABA, and methyl jasmonate (MeJA), followed by gibberellic acid (GA), auxin, and SA (Figure 3 and Supplementary Table S7). Notably, the promoter regions of 163 *NtPOD* genes contained cis-acting elements related to ABA (ABRE) and 121 genes were involved in MeJA response elements (TGACG-motif). These results indicate that the *NtPOD* family may be involved in the complex hormone regulatory network. Furthermore, the elements regulating plant development had 6 elements: seed, root, endosperm, palisade mesophyll cells, and meristem-specific expression elements. Moreover, a number of site-binding related elements were also identified in the promoter regions of *NtPODs* (Figure 3 and Supplementary Table S7). Hence, diverse cis-elements among *NtPOD* genes may reflect their potential functional variation.

**FIGURE 4:**
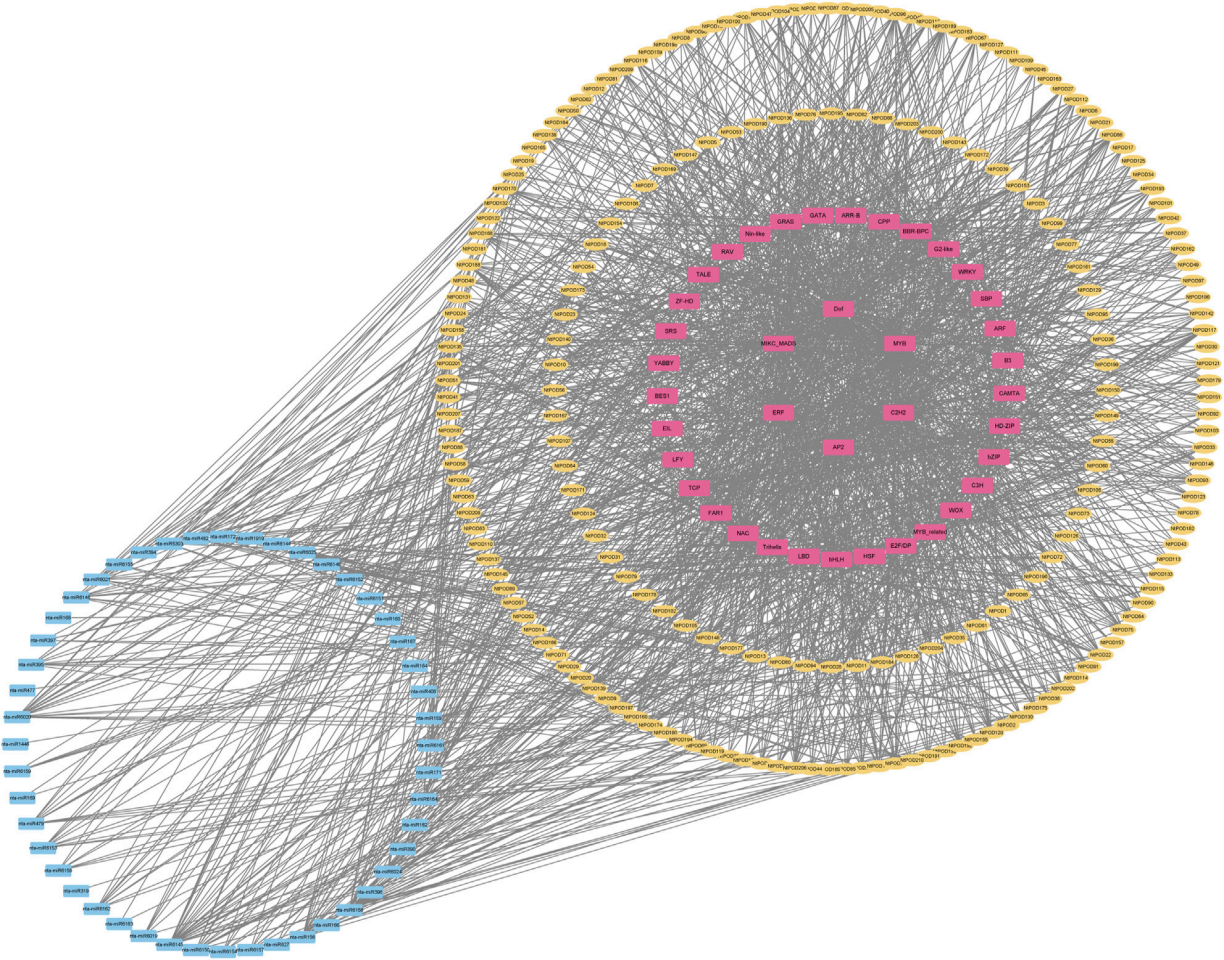
Regulation network of *NtPODs* in tobacco. The yellow, pink, and blue boxes represent *NtPOD* genes, TFs, and miRNAs, respectively. The black line represents the interaction.

Due to abundant cis-elements enriched in the promoter regions of NtPOD genes, we speculated that the corresponding transcription factors (TFs) may directly regulate *NtPOD* genes in tobacco. Therefore, we explored the regulatory relationship between transcription factors and *NtPODs* using PlantTFDB (Jin et al., 2016). In total, 349 TF members from 39 families may play important roles in the regulation of *NtPODs* (Figure 4). Among these, MIKC_MADS, Dof, MYB, AP2 and C2H2 transcription factors were the most abundant. We also investigated potential miRNA-binding sites for *NtPODs* using PsRNATarget (Dai and Zhao, 2011). Finally, 49 miRNA families, consisting of 129 miRNAs, may have regulatory relationships with *NtPODs* (Figure 4 and Supplementary Table S8). Most miRNAs had several *NtPOD* targets, including nta-miR6156 which could target 26 *NtPOD* genes (Figure 4 and Supplementary Table S8). In contrast, some *NtPODs* could be targeted by several miRNAs. For example, *NtPOD102* can be targeted by several miRNAs, including miR166 and miR168 (Figure 4 and Supplementary Table S8). The relationship between *NtPODs* and TF/miRNAs requires further study.

### Expression Analysis of *NtPOD* Genes in Eight Representative Tissues

**FIGURE 5:**
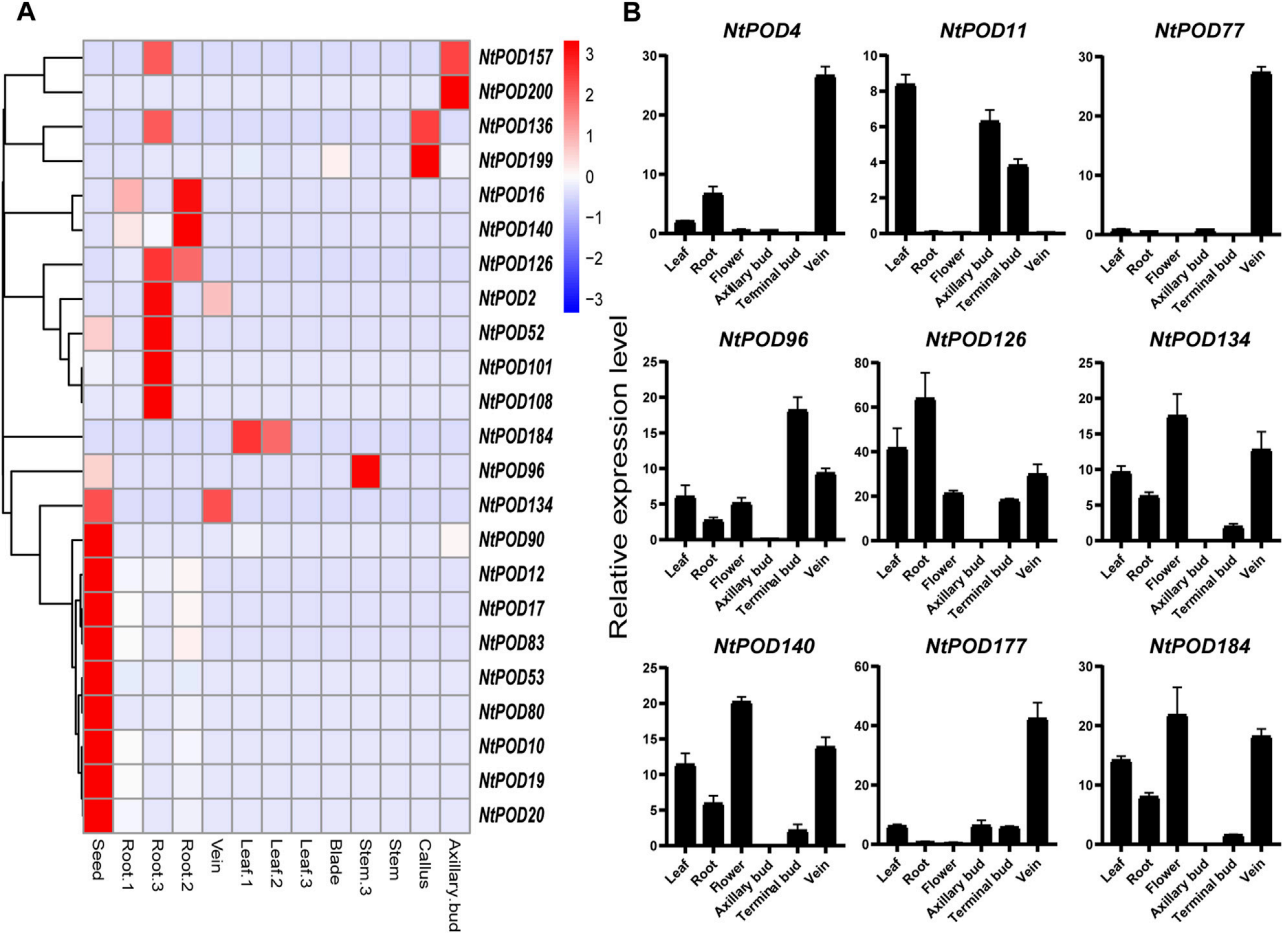
Expression profiles of tissue-specific *NtPOD* genes in different tissues. **(A)** Heatmap of the expression in different tissues at three development stages [seedling (1), mature (2), and 2 days after topping (3)], scaled by rows. **(B)** qRT-PCR analysis of nine selected *NtPODs* in axillary bud, flower, leaf, root, terminal bud, and vein.

To further study the potential function of each *NtPOD* gene, the expression patterns in eight tissues (root, stem, leaf, blade, vein, axillary bud, callus, and seed) were explored. Except for 21 *NtPODs* that not or weakly expressed in the eight tissues, the remaining 189 genes were expressed in at least one tissue (Supplementary Figure S4 and Supplementary Table S9). As shown in Figure 5A, a number of *NtPOD*s exhibited distinct tissue-specific expression patterns. It is worth noting that the number of *NtPOD* genes expressed in the roots was the highest, suggesting that most *NtPOD* genes play important roles in the root (Supplementary Figure S4). Interestingly, several *NtPOD* genes, such as *NtPOD12* and *NtPOD90*, were specifically expressed in the seed (Figure 5A). Furthermore, *NtPOD199* may play an important role in callus redifferentiation due to its high expression in callus. Nine *NtPOD* genes (*NtPOD4*, *11*, *77*, *96, 126*, *134*, *140*, *177*, and *184*) with tissue-specific expression were randomly selected for further qRT-PCR analysis in different tissues, and the results were similar to those of the RNA-seq analyses (Figure 5B). *NtPOD126* and *NtPOD4* showed higher relative transcription levels in the roots and veins, respectively. Consistent with phylogenetic and motif analyses, the specific and varied expression profiles of *NtPOD* genes in different tissues also suggested their diverse roles. Moreover, the expression of *NtPODs* also varied in three developmental stages (seedling, mature, and 2 days after the topping) (Figure 5A and Supplementary Figure S4). For the genes expressed in leaves, six (*NtPOD27*, *78*, *79*, *98*, *115* and *206*) showed relatively stronger expression during seedling stages, suggesting that these genes may play specific roles during the early stages of leaf development. However, the other six (*NtPOD2*, *52*, *75*, *101*, *108*, and *157*) and five (*NtPOD69*, *72*, *158, 192*, and *201*) genes were relatively higher in roots and leaves during the 2 days after the topping stage, respectively.

### Expression Analysis of *NtPOD* Genes in Response to Various Abiotic and Biotic Stresses

**FIGURE 6:**
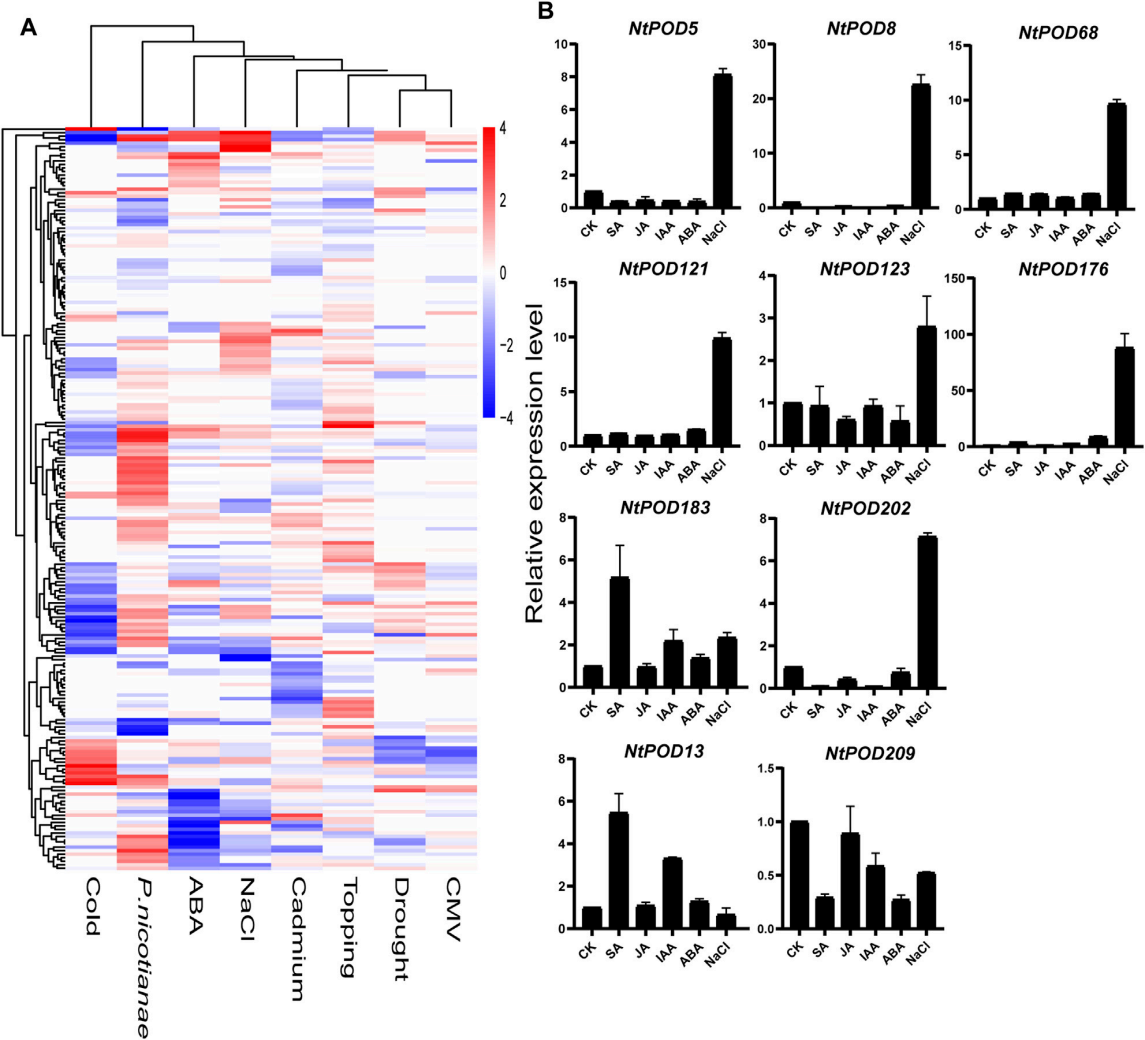
Expression patterns of *NtPOD* genes under various stress treatments. **(A)** Expression changes of *NtPOD* genes under cold, ABA, NaCl, cadmium, topping, *P. nicotiana*, drought, and CMV stresses. The expression change is indicated by the ratio of FPKM value of the treatment to that of the control (CK). **(B)** Results of qRT-PCR analysis of ten *NtPOD* genes in response to NaCl and hormones (ABA, IAA, SA, JA) treatment.

To further explore the response of *NtPODs* during various stress responses, the expression patterns of all *NtPODs* were investigated using publicly available transcriptome data (Supplementary Table S10). As shown in Figure 6A, three genes (*NtPOD68*, *121*, and *192*) were significantly up-regulated after salt treatment. Some *NtPOD* genes were extremely sensitive to the cold stress and exhibited significant down-regulation, such as *NtPOD86*, *92*, *104*, *136*, *154*, *155*, and *166*. In addition, ABA treatment significantly reduced the expression of 11 *NtPODs*. Among the various biotic stresses, 59 *NtPODs* showed a significant response upon inoculation with *P. nicotianae*, whereas only a few genes changed slightly under CMV treatment. Interestingly, most *NtPODs* were specifically involved in individual stress treatment rather than a universal response. To compare the transcription of *NtPOD* genes between different hormone treatments including ABA, IAA, SA, and JA, we selected eight significantly up-regulated (*NtPOD5, 8, 68, 121*, *123*, *176, 183 and 202*) and two significantly down-regulated gene (*NtPOD13* and *209*) under salt stress to further explore the effects of various plant hormone treatments by qRT-PCR (Figure 6B). Five genes (*NtPOD5*, *8*, *68*, *121*, *176* and *202*) were found to be extremely sensitive to salinity. Their expression levels increased more than 7-fold compared to the control. Consistent with the RNA-seq analysis, the expression level of *NtPOD13* and *NtPOD209* decreased after salt stress. Under ABA treatment, *NtPOD176* were up-regulated, whereas *NtPOD8* and *NtPOD209* were down-regulated. However, the expression levels of *NtPOD13*, *176* and *NtPOD183* increased slightly upon SA and IAA treatment, whereas JA treatment induced the expression of *NtPOD202* and *NtPOD209*. In summary, these results demonstrate that *NtPOD* genes are involved in various abiotic and biotic stress treatments.

### Subcellular Localization Analysis and 3D Structure Prediction of NtPOD Proteins

**FIGURE 7:**
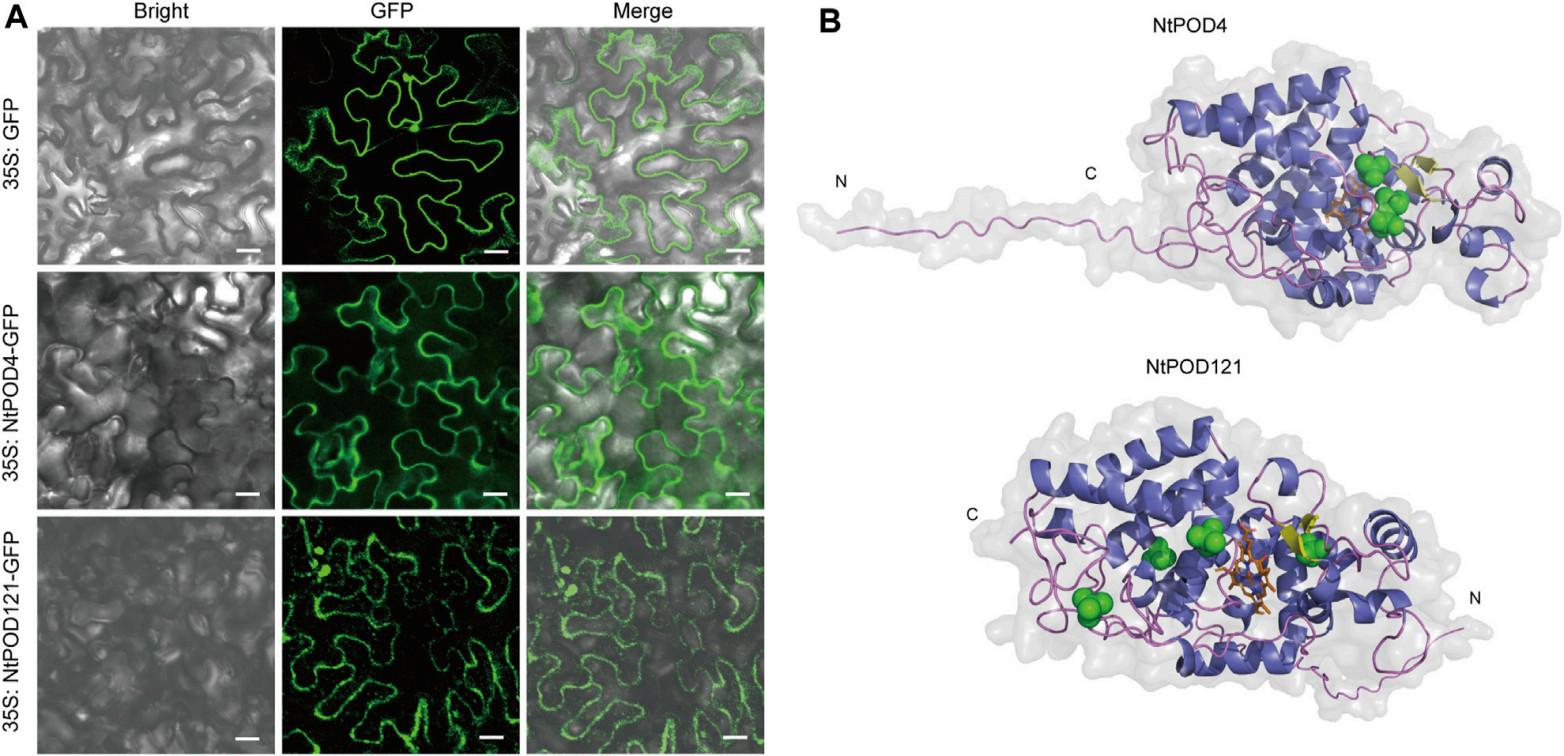
Subcellular localization and 3D structure of NtPOD4 and NtPOD121. **(A)** Subcellular localization of NtPOD4 and NtPOD121. Scale bars: 20 µM. **(B)** Putative 3D structures of two class III peroxidases: POD4 (predicted location in plasma membrane) and NtPOD121 (predicted location in chloroplast). Colors indicate secondary structures: α-helixes (blue), β-sheets (yellow), loops (purple), disulfides (green), and heme group (orange).

The analysis based on the NtPOD amino acid sequences indicated 162 (77.1%) NtPODs contain an N-terminal signal peptide with a putative cleavage site (Supplementary Table S4). After the identification of peroxidase sequences with transmembrane domains, NtPODs were classified as 119 secreted and 91 membrane-bound peroxidases (Supplementary Figure S5A). Furthermore, 91.2% (83) of the membrane-bound peroxidases contained N-terminal signal peptides (Supplementary Table S4). In addition, the majority of NtPODs were predicted a chloroplast location (n = 92) and extracellular regions (*n* = 63) (Supplementary Figure S5B). Subcellular localization prediction revealed that NtPOD4 contained a putative signal peptide with a predicted plasma membrane (PM) location, whereas NtPOD121 contained a putative signal peptide with a predicted chloroplast location (Supplementary Table S4). However, result of subcellular localization assay using transient expression of NtPOD4-GFP and NtPOD121-GFP in *Nicotiana benthamiana* leaves, suggested both the plasma membrane localization for these two peroxidases (Figure 7A).

In order to gather additional information about the NtPOD proteins, the structural models of eight peroxidases were built using Alphafold2 (Supplementary Figure S6). The ERRAT test scores by the SAVES server for the eight NtPOD 3D models above 88, which indicated that the predicted 3D models were reliable. Our results showed that NtPOD4 and NtPOD121 were monomers that contained two β-sheets with 17 (NtPOD4) and 20 (NtPOD121) α-helices. Similarly, both NtPOD4 and NtPDO121 had characteristic loops at their N-termini (Figure 7B). As the classical peroxidase structure, NtPOD121 and NtPOD4 contain eight Cys residues, which are necessary for the formation of four conserved disulfide bridges.

## Discussion

Environmental pressure poses a considerable challenge to crop production. Class III peroxidases are widely distributed among terrestrial plant species and play an essential role in plant resistance to different stresses, such as salt, drought, and metal toxicity (Almagro et al., 2009). Our study is the first comprehensive and systematic report to characterize the *NtPOD* gene family in *N. tabacum*. PODs encoded by large multigene family have been found in many plants; however, the number of PODs varies greatly among different plants (Kidwai et al., 2020). In total, 210 class III peroxidase genes were identified in the tobacco genome. This number is larger than that of most reported species, such as *Arabidopsis* (73) (Tognolli et al., 2002), rice (138) (Passardi et al., 2004), maize (119) (Wang Y et al., 2015), soybean (124) (Aleem et al., 2022), polar (93) (Ren et al., 2014), Chinese pear (94) (Cao et al., 2016), carrot (102) (Meng et al., 2021), and other diploid genome plants, but fewer than that in hexaploid wheat (374) (Su et al., 2020). This indicates a considerable expansion of the POD gene family in tobacco and wheat compared to other plant species. In plants, gene family expansion is typically the result of polyploidy and gene duplication. Tobacco (*N. tabacum*) is an allotetraploid (2n = 48) that originates from chromosome doubling after an interspecific hybridization event between *N. tomentosiformis* (2n = 24) and *N. sylvestris* (2n = 24), which occurred nearly 200,000 years ago (Lim et al., 2004). Tobacco POD genes may undergo duplication along with the whole genome duplication event. Gene duplication can help organisms adapt to various environmental conditions (Kondrashov, 2012). Gene family expansion primarily occurs via three modes: segmental duplication of multiple genes, tandem duplication of individual genes, and whole-genome duplication (Panchy et al., 2016). We identified 103 duplication events in *NtPOD* gene family, involving 109 paralogs. Segmental duplication is thought to be the main driver of *NtPOD* evolution, as 71.6% (78) of the duplications were segmental events (Supplementary Table S6). *Ka* and *Ks* analyses revealed that the evolution of POD genes was driven mainly by purifying selection and that only a few POD sequences may have experienced positive selection during the evolutionary period. Similar results were found in the study on the evolution of PODs in maize (Wang Y et al., 2015), sweet orange (Li et al., 2020), while in contrast with results in soybean (Aleem et al., 2022), *Arabidopsis* (Tognolli et al., 2002), and rice (Passardi et al., 2004). Based on phylogenetic relationships, the *NtPOD* family was categorized into six subgroups. Notably, subgroup VI was characterized by multiple exons and different motifs from the other five subgroups, which was consistent with previous studies in cassava (Wu et al., 2019), Chinese pear (Cao et al., 2016), and watermelon (Yang et al., 2022). Therefore, we inferred that POD subgroup VI may have similar origins with other plants and have some specific functions in plants.

Peroxidases can be classified into secreted and membrane-bound peroxidases. Some are secreted into the extracellular space and cell walls under the guidance of N-terminal signal peptides, while some membrane-bound peroxidases are targeted to the ER, vacuole, plasma membrane, or thylakoid (Ren et al., 2014; Lüthje and Martinez-Cortes, 2018). Correspondingly, our results showed that NtPODs were located in the extracellular space, PM, and intracellular spaces, including the thylakoid, cytoplasm, ER, and nucleus. In this study, 43% of the NtPODs were considered as membrane-binding proteins because their N-terminus contained transmembrane domains, which is consistent with previous reports (Lüthje and Martinez-Cortes, 2018). The presence of a cleavable signal peptide at the N-terminus, which varies in cleavage sites from 14 to 34 residues, indicated the secretory nature of 162 NtPODs. Interestingly, members of subgroup VI were predicted to be localized in the cytoplasm, endoplasmic reticulum, and chloroplast, and all lacked N-terminal signal peptides. However, the subcellular localization result showed that NtPOD4 (predicted location in plasma membrane) and NtPOD121 (predicted location in chloroplast) was both localized to the plasma membrane, inconsistent with the predictions. Due to limitation of prediction tools/methods, inconsistent results for subcellular location between prediction and experiment validation are widespread (Nielsen, 2015). Hence, more experimental work might need to explore whether there were some specific domains or structures for specific NtPOD.

The structure of class III peroxidases is well-conserved (Welinder et al., 2002; Mathé et al., 2010). These proteins contain N-terminal signal peptides, binding sites for heme and calcium, and eight conserved Cys residues required for four conserved disulfide bridges (Welinder et al., 2002). The 3D structure of a protein is important for understanding its detailed functional mechanism. Deriving the protein 3D structure could facilitate a mechanistic understanding of POD function. Hence, we attempted to predict the 3D structures of NtPODs using AlphaFold2 (Supplementary Figure S6). The results revealed the diversity of their N-terminal structures, which may explain their different subcellular localizations. Although Class III peroxidases share structural features, which indicated the likelihood of similar mechanisms, the high degree of similarity in protein structure was relatively inconsistent with the functional diversity of these enzymes. These data support the idea that, due to the low specificity of class III peroxidase substrates, the response to specific environment may be the key to determine the role of individual peroxidase isomer in plants.

In plants, a complex gene regulatory network consists of transcription factors, regulatory RNAs, and enzymes that regulate plant growth and development (Ibraheem et al., 2010; Chen et al., 2018). Transcription is initiated by the interaction of TFs, which usually combine with cis-acting regulatory elements in genes in response to environmental changes (Priest et al., 2009). An analysis of the cis-elements in *NtPOD* gene promoters resulted in the detection of six major types of cis-elements associated with biotic/abiotic stress, hormone response, light response, and developmental processes. Based on these cis-elements, we found 349 TF members from 39 families might play important roles in the regulation of *NtPODs* (Figure 4). Many TF families implicated in stress responses have been identified, including WRKY (Niu et al., 2012), MYB (Chen et al., 2015), NAC (Nakashima et al., 2007), and bZIP (Hsieh et al., 2010). Moreover, identifying the potential target sites of miRNAs provides valuable insights into the biological functions of miRNAs and target genes related to plant growth and development, stress response and adaptation, and signaling mechanisms. Finally, 49 miRNA families, consisting of 129 miRNAs, may have regulatory relationships with *NtPODs* (Figure 4 and Supplementary Table S8). Among this regulatory network, *NtPOD93* could be targeted by miR172, which was one of well-known miRNAs involved in drought response (Han et al., 2013). Meanwhile, one well-known drought response TF, Dof (Li et al., 2021), might also regulate *NtPOD93* (Figure 4). Another example was miR395-NtPOD202-AP2 module, and many evidences suggested that both miR395 (Kim et al., 2010; Çakır et al., 2021) and AP2 (Zhang et al., 2009) might play important roles in salt treatment. Hence, the POD interaction network constructed in our study provides insights into the regulation of peroxides in plants in response to various stresses.

Peroxidases are involved in cell wall-related reactions, metabolic pathways, and stress-related processes (Veljović Jovanović et al., 2018). These enzymes play key roles in the scavenging of ROS (Das and Roychoudhury, 2014). Although it is challenging to study individual POD genes owing to their functional redundancy and low substrate specificity (Cosio and Dunand, 2009), accumulating evidences suggest that the overexpression of POD genes results in increased plant tolerance to stresses. To understand the potential functions of the *NtPOD* genes in stress resistance, public RNA-seq data were used to investigate their expression patterns. The complexity and diversity of *NtPOD* expression patterns under various biotic and abiotic stresses were observed. In tobacco, the expression levels of only few POD genes increased after drought, cold, salt, topping, and cadmium treatments, while the expression levels of many members of the POD family increased after *P. nicotianae* infection. Meanwhile, individual POD genes are usually sensitive to one specific external stress, and few genes are widely responsive to various biotic and abiotic stresses. For example, *NtPOD166* was responsive only to cold treatment. *NtPOD93* was localized in vacuoles and induced only by drought stress. Moreover, the regulatory network constructed in our study implied that *NtPOD93* may be regulated by miR172 during drought response. *NtPOD131* was located in the chloroplast and associated with Cd stress. Salinization stress is one of the environmental factors that limit tobacco yield and quality, and some plant hormones, such as ABA, IAA, SA, and JA, are considered as salt stress response hormones (Yu et al., 2020). The qRT-PCR results were consistent with the RNA-seq gene expression patterns. Salt treatment resulted in increased transcript levels of *NtPOD5, 8, 68, 176, 121,* and *202*, while the expression level of *NtPOD209* and *NtPOD13* was decreased. *NtPOD68* and *NtPOD121* significantly responded to hormone signals, such as ABA, IAA, SA, and JA, suggesting that these hormones exhibit complex signaling regulation to control plant responses to salt stress. *NtPOD5, 8, 68, 176, 121,* and *202* could be regarded as candidates that participate in the salt stress response. However, more work need to be performed to investigate the detailed mechanism for these candidates. In summary, the expression patterns of *NtPODs* under various abiotic and biotic stresses are complex and diverse, and may be related to the functional diversity of POD gene family members. These results provide useful insights into the potential capabilities of *NtPODs* under various abiotic and biotic stresses.

## Conclusion

In the present study, we identified, characterized, and analyzed the members of the class III peroxidase family in tobacco by investigating phylogeny, protein properties, 3D models, and expression patterns. Many *NtPOD* genes were found to be expressed in a tissue-specific manner, with showing involvement in specific biotic and abiotic stresses. The function of these *NtPOD* genes is of great significance for the improvement of resistance to stresses in tobacco plants, and will need to be elucidated further in future studies.

## Data Availability

The datasets presented in this study can be found in online repositories. The names of the repository/repositories and accession number(s) can be found in the article/Supplementary Material
