# Supplementary material for: Genome-Wide Identification and Analysis of the Class III Peroxidase Gene Family in Tobacco (Nicotiana tabacum)
Source: Front Genet. 2022 Jun 13;13:916867. doi: 10.3389/fgene.2022.916867 (PMC9234461; doi:10.3389/fgene.2022.916867)
Supplement: Supplementary file 1 [file DataSheet1.docx]

Supplementary Figures


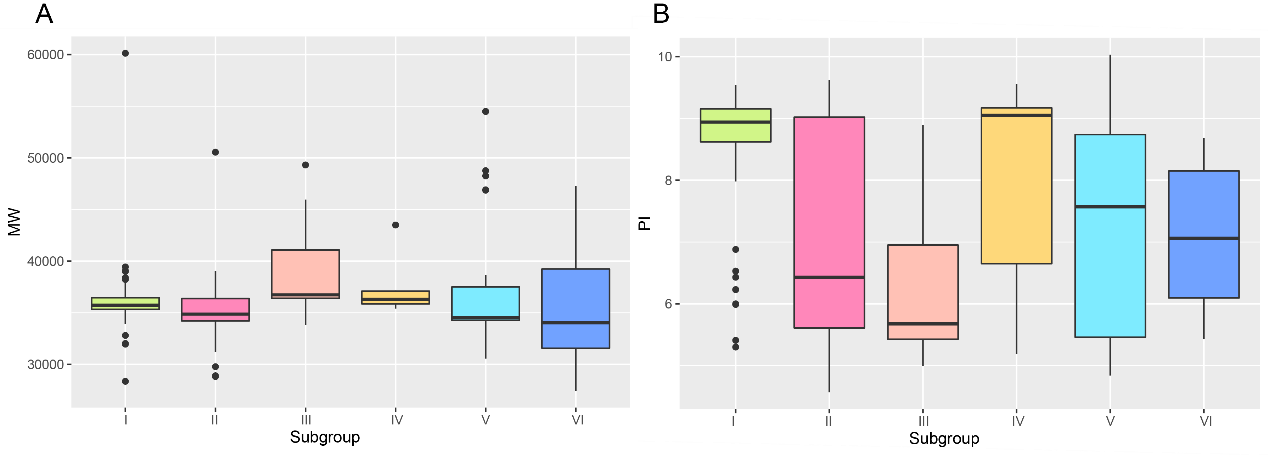


**Supplementary Figure 1.** Box plot for molecular weight (A) and Isoelectric point (B) of six different phylogenetic groups (I–VI) of NtPODs.


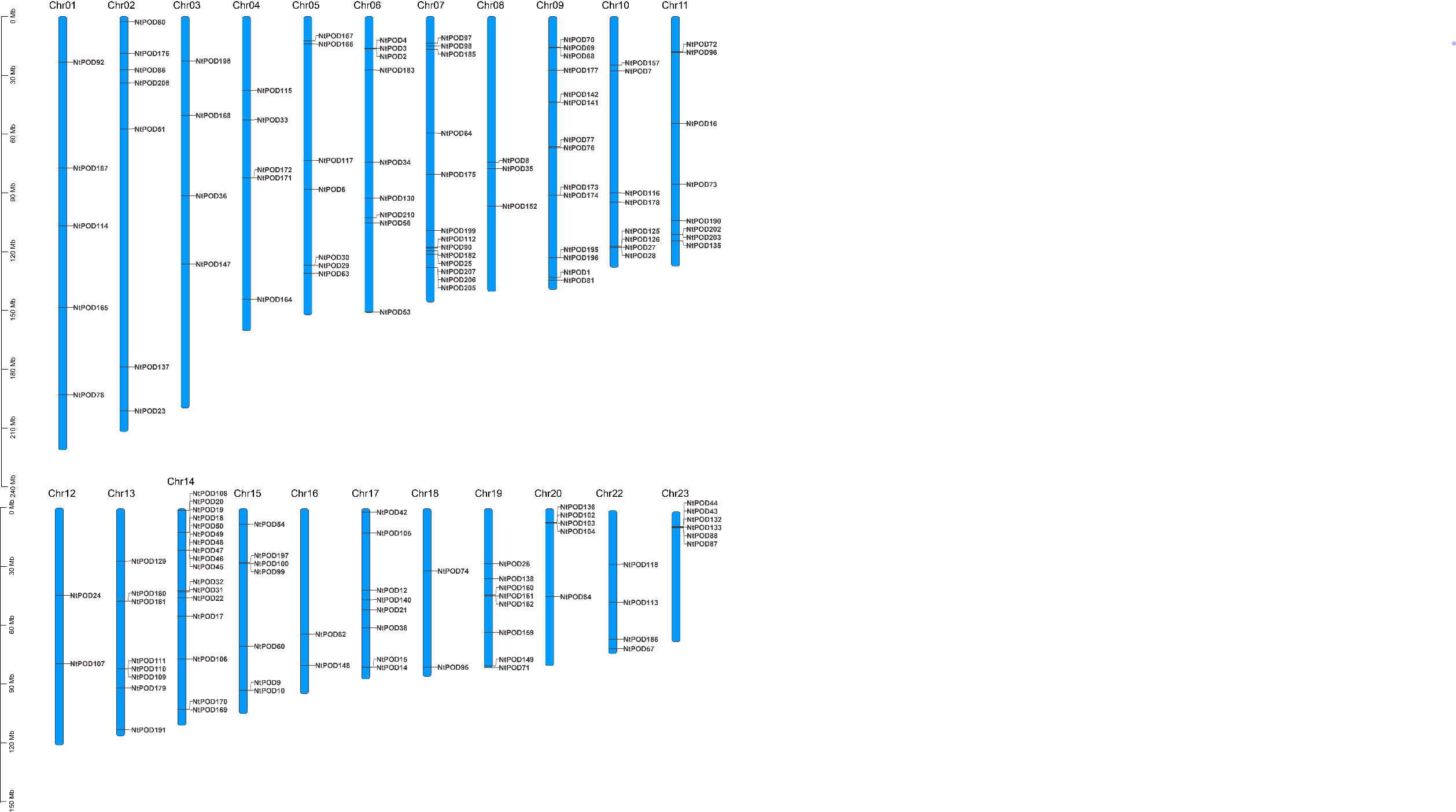


**Supplementary Figure 2.** Chromosome distribution of 210 *NtPODs* on tobacco chromosomes.


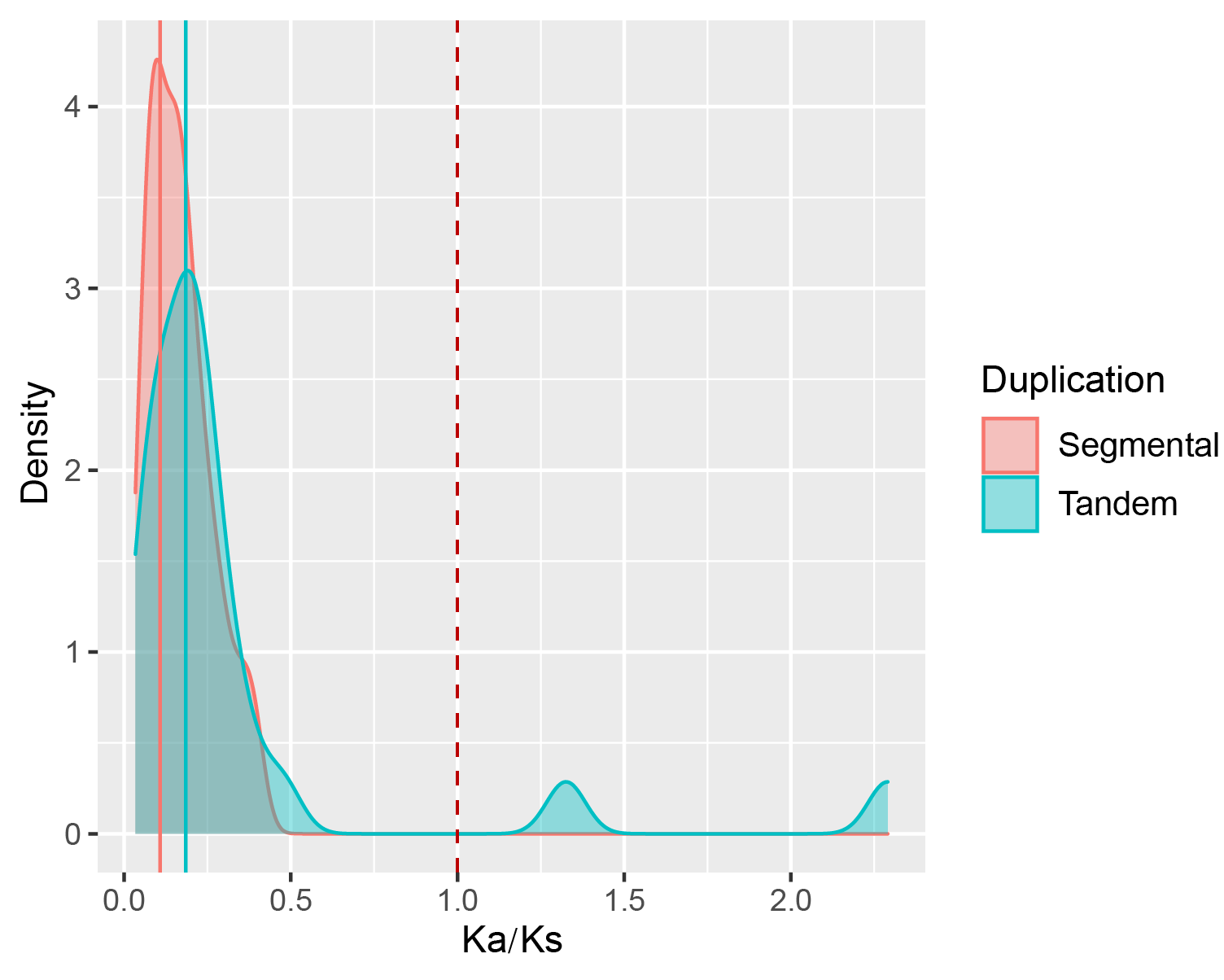
**Supplementary Figure 3.** Frequency distribution of *Ka/Ks* ratios of *NtPODs* among tandem and segmental duplicated paralogous genes.

**
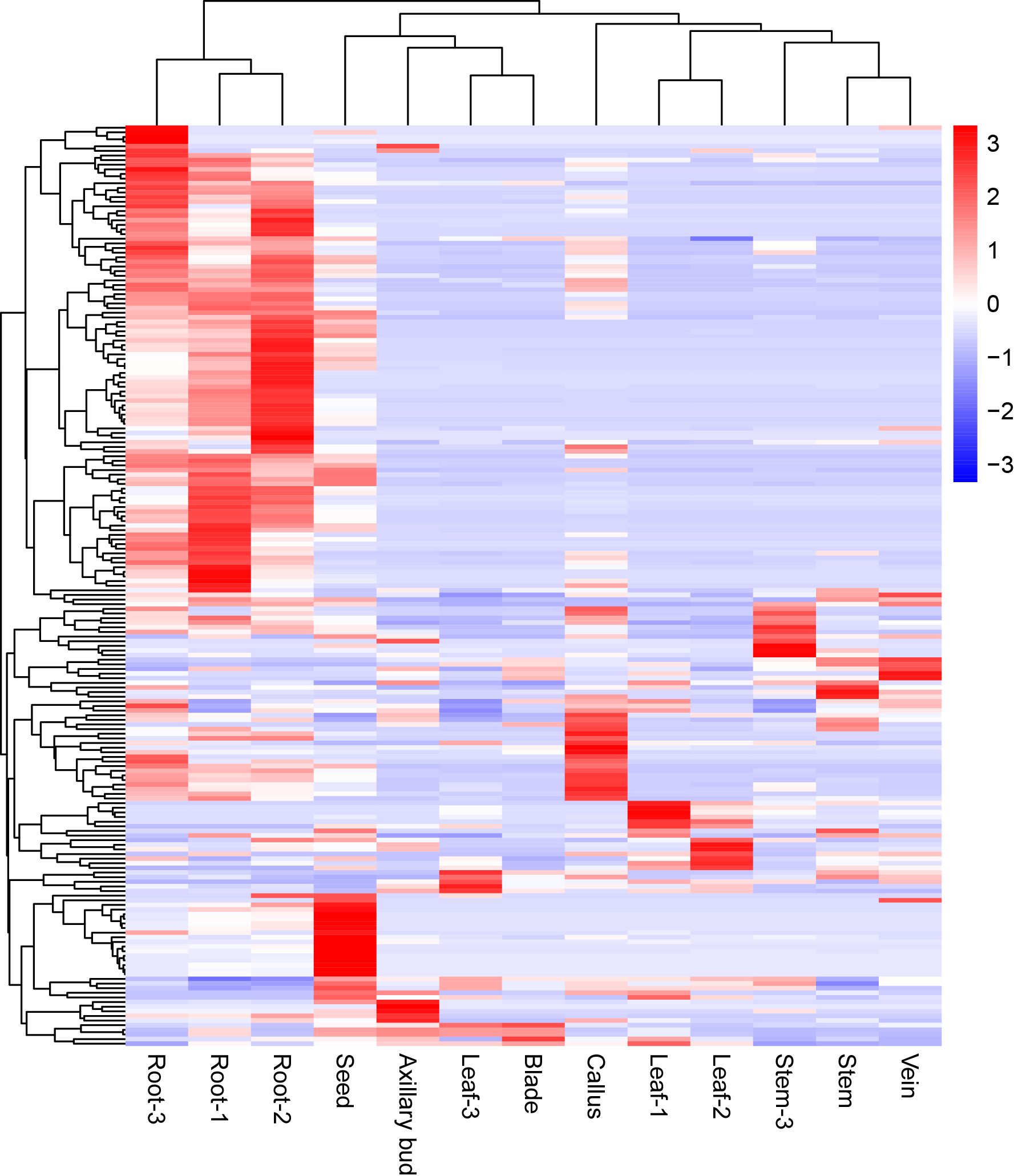
Supplementary Figure 4.** Heatmap of *NtPOD*s gene expression in different tissues at 3 developmental stages (seedling (1), mature (2), and two days after topping (3)).


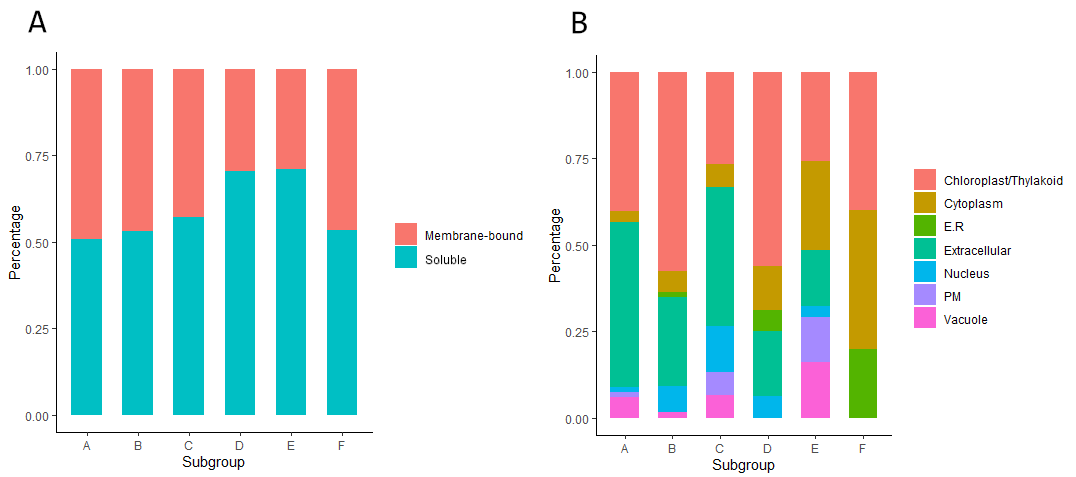


**Supplementary Figure 5.** Percentage of membrane-bound and soluble PODs (A) and subcellular localization (B) of six different phylogenetic groups (I–VI) of NtPODs.


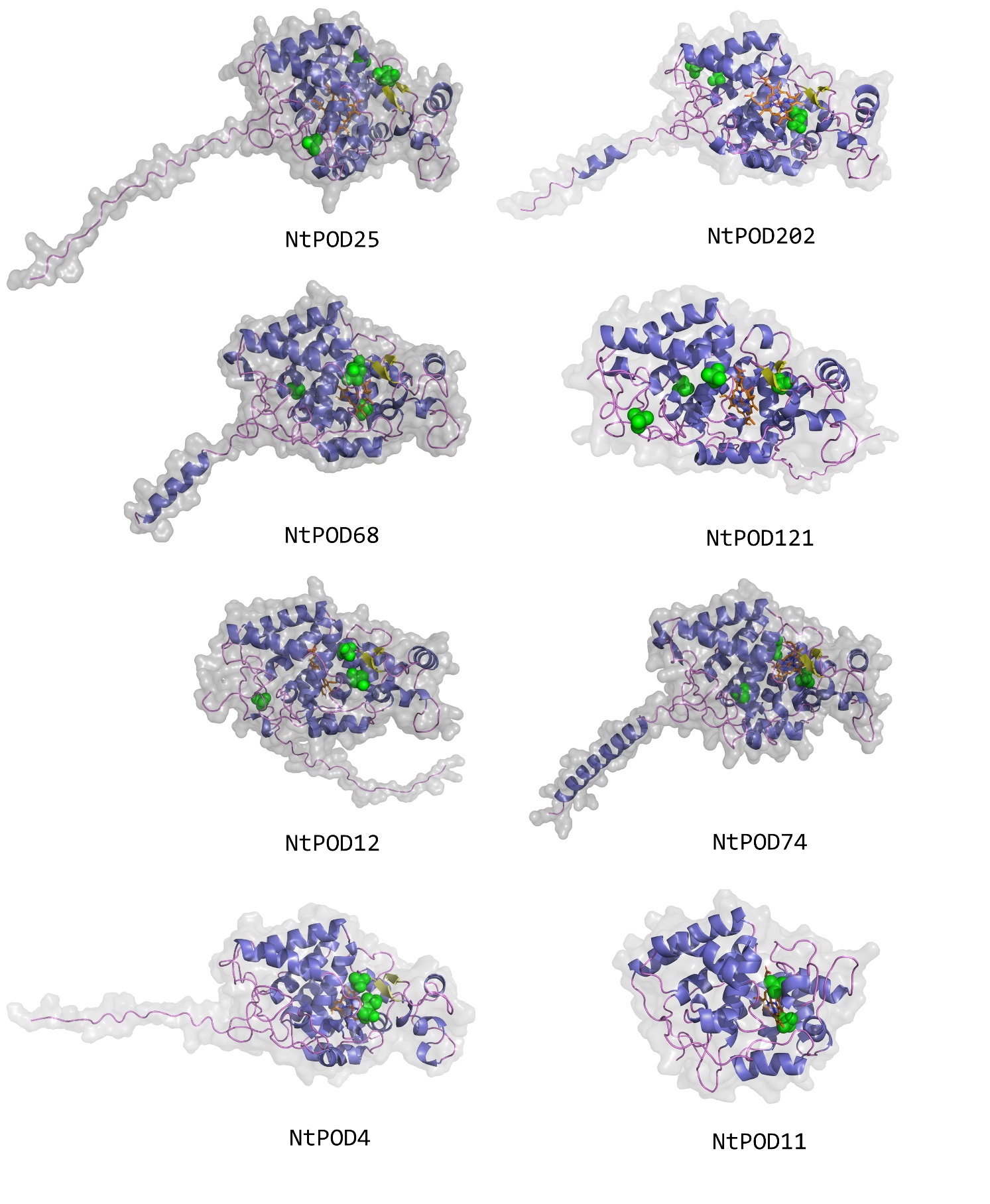
**Supplementary Figure 6.** The predicted 3D structure model of putative soluble (NtPOD25, 202, 68, 121, 12) and putative membrane-bound NtPODs (NtPOD74, 4, and 11). Colors indicate secondary structures: α-helixes (blue), β-sheets (yellow), loops (purple), disulfides (green), and heme group (orange).
